# Psychological Impact During the First Outbreak of COVID-19 in Italy

**DOI:** 10.3389/fpsyt.2020.559266

**Published:** 2020-11-02

**Authors:** Roberta Ferrucci, Alberto Averna, Daniela Marino, Maria Rita Reitano, Fabiana Ruggiero, Francesca Mameli, Michelangelo Dini, Barbara Poletti, Sergio Barbieri, Alberto Priori, Gabriella Pravettoni

**Affiliations:** ^1^CRC Aldo Ravelli, Dipartimento di Scienze della Salute, Università degli Studi di Milano, Milano, Italy; ^2^III Clinica Neurologica, ASST-Santi Paolo e Carlo, Presidio San Paolo, Milano, Italy; ^3^U.O. di Neurofisiopatologia, Fondazione IRCCS Ca' Granda Ospedale Maggiore Policlinico, Milano, Italy; ^4^Servizio di Neuropsicologia e Psicologia Clinica, IRCCS Istituto Auxologico Italiano, Milano, Italy; ^5^Dipartimento di Oncologia ed Emato-Oncologia, Università degli Studi di Milano, Milano, Italy; ^6^Divisione di Psicooncologia, Istituto Europeo di Oncologia, Milano, Italy

**Keywords:** anxiety, fear, sexuality, psychological distress, COVID-19

## Abstract

The first outbreak of COVID-19 in Italy was confirmed on February 21, 2020. Subsequently, COVID-19 turned into a global pandemic, causing a global health emergency, triggering an unprecedented event in the modern era. This study assessed the immediate psychological impact of the COVID-19 epidemic on emotional health and well-being.

An *ad hoc* questionnaire was designed for online completion to expedite data collection during the COVID-19 outbreak. People were invited to participate in the study via social media and email from 4 to 18 March 2020. The entire survey comprised of 21 questions, covering a wide range of factors, such as demographics, disease knowledge, psychological impact, daily life activities, and psychological precautionary measures. The main outcome measure was psychological impact. This was measured based on intensity and prevalence of self-reported feelings of anxiety, fear, sadness, anger, and concern during the epidemic.

In total, 10,025 respondents completed the online survey. Of these, about 73% were females, and 100% of the sample possessed good knowledge of the disease. The greatest prevalence of high psychological impact was reported in the <34 years' age group and in north Italy. Additionally, the psychological impact influenced important daily life activities, such as sexuality and nutrition.

Our study provides information about the immediate psychological (emotional feelings) responses of Italy's general population to the COVID-19 epidemic. The survey covers several factors that can influence mental health; our results help gauge the psychological burden on the community and offer ways to minimize the impact.

## Introduction

COVID-19 (coronavirus disease 2019; the pathogen called SARS-CoV-2; previously 2019-nCoV) is an acute and highly contagious viral disease which can cause rapidly spreading outbreaks of respiratory diseases (1). It was first diagnosed in Wuhan, China. Following this, it first spread to Italy, then to other European countries, and eventually, throughout the globe, affecting 184 countries from December 2019 to April 8, 2020. (2). Governments worldwide are under increased pressure to stop the outbreak from spiraling into a global health emergency.

Italy's first outbreak of COVID-19 was confirmed on February 21, 2020. In the beginning, it rapidly spread to north Italy and then affected all other regions. This soon became a global pandemic (WHO) (2), causing a global health emergency, triggering an unprecedented event in the modern era.

Disease control procedures focusing on restraining the virus were put in place across all regions, including quarantine, movement restrictions, military control, and bio-security measures.

Color-coded COVID-19 control zones were established within the first 2 weeks of the outbreak based on the level of risk of the virus spreading. These zones were re-assigned with the spread of the disease in the area, and each zone was subject to specific controls and restrictions. Currently, more than 139.422 people are infected, ~80% in the north and at least 20% in south-central. Current data from disease surveillance and monitoring indicate the presence of active infection in Italy. When Italy will be declared COVID-19-free remains uncertain.

The impact on people was both economic, through financial and business losses and psychological, through the loss of freedom during quarantine and exposure to media images on television and in newspapers (3–5). Repeated media exposure can increase anxiety and heighten stress responses, this negatively affects health. Further, misplaced health-protective and help-seeking behavior can overburden health care facilities and available resources (4, 6). During Ebola and H1N1 outbreaks, media coverage of events had unintended consequences for those at a relatively lower risk of direct exposure, leading to severe public health repercussions. The need to combat false information and rumors is extremely crucial in the age of social media and information that can go viral (7).

Although several COVID-19-related research is emerging, few so far focused on the psychological impact on people directly exposed to such outbreaks (8–11).

The existing studies analyzed factors related to symptoms of anxiety and anger after quarantine. Brooks et al. reported that individuals with a greater knowledge about the disease during the initial stages of the MERS outbreak experienced increased anxiety and had greater trust in unofficial information (3). Wang et al. reported that during the initial phase of the COVID-19 outbreak in China, more than half the respondents rated the psychological impact as moderate-to-severe and about one-third reported to have experienced moderate-to-severe anxiety (10).

This study assessed the immediate psychological impact of the COVID-19 epidemic on emotional health and well-being.

## Methods

### Survey Design and Sampling

An *ad hoc* questionnaire was designed for online completion to expedite data collection during the COVID-19 outbreak. People were invited to participate in the study via social media and email. The procedure involved filling an online consent form. All data were collected anonymously and stored in a password-protected electronic format.

More than 10,000 emails were sent to individuals as well as associations, clubs, and Facebook groups, with the assumption that the information would be forwarded within their own social circles, nationally. The initial invitation to participate was sent on March 4, 2020 (Week 2 of the outbreak). The survey remained open until March 18, 2020 (Week 4 of the outbreak), and date of completion was recorded with each respondent's data.

### Survey Content and Outcome Measures

The questionnaire assessed the self-reported psychological impact. The content was reviewed by a small group of public mental health professionals (clinical psychologists). The entire survey comprised of 21 closed questions, covering a wide range of factors, such as demographics, disease knowledge, psychological impact, daily life activities, psychological precautionary measures, and frightening events (Refer to Table 1). The demographic information included: gender, age, highest level of educational qualification, and region of residence.

**Table 1:** Demographic characteristics of the sample.

		***N***	**%**
**Gender**	Male	2,741	27.34
	Female	7,284	72.65
**Age category**	≤34	3,765	37.55
	35–64	5,816	58
	≥65	444	4.42
**Education level**	5–8 anni	163	1.62
	8–13 anni	735	7.33
	13–17	2,110	21.04
	>17	7,017	70
**Regions of Italy**	Abruzzo	58	0.58
	Aosta valley	6	0.06
	Apulia	295	2.94
	Basilicata	35	0.35
	Calabria	75	0.75
	Campania	148	1.48
	Emilia Romagna	476	4.75
	Friuli Venezia Giulia	88	0.88
	Lazio	1,046	10.43
	Liguria	248	2.47
	Lombardy	5,237	52.23
	Marche	93	0.93
	Molise	65	0.65
	Piedmont	694	6.92
	Sardinia	138	1.37
	Sicily	140	1.39
	Trentino South Tyrol	42	0.42
	Tuscany	552	5.50
	Umbria	57	0.57
	Veneto	430	4.29
	Not reported	29	0.29
	Nationals living abroad	73	0.73

The main outcome measure reported in this study was psychological impact. This was measured based on intensity and prevalence of self-reported feelings of anxiety, fear, sadness, anger, and concern during the pandemic. This measure was assessed using questions that inquired about the intensity with which the respondents experienced certain feelings/emotions during the epidemic period. The responses were scored on a four-point scale, depending on the intensity of each emotion experienced (0 = “none” and 4 = “very high” intensity).

Psychological impact category included: self-reported feelings of anxiety, fear, sadness, anger, and concern for well-being (*What emotions do you experience after receiving information about COVID-19?*).

Daily life activities included: nutrition, sleep, sexuality, relationship with others, and sense of freedom (*How much does the current situation negatively affect the following?...*).

Psychological precautionary measures included: listening to less drastic media information, psychotherapy, use of disinfectants, use of medical device, and avoiding crowded places (*What would make you feel safer in this period?*).

Frightening events included: falling sick, economic crises, job loss, death, psychosis, and quarantine (*What scares you about COVID-19?*).

### Statistical Methods

Descriptive statistics were used to calculate categorical variables. Percentage of responses was calculated according to the number of respondents per response with respect to the total number of responses to a question.

We performed logistic regression using the MATLAB command “mnrfit” to consider the relationship between factors of age, sex, and region and the four-point scale used to rate the endpoints (i.e., none, low, moderate, and high).

The association between ranked scores for questions were assessed using Kendall's Tau, a non-parametric correlation for discrete scores. Tau-b corrects for the presence of ties and also has a range between −1 and 1. Kendall's tau b of at least 0.7 represents a very strong relationship; 0.4–0.699, a strong relationship; 0.3–0.399, a moderate relationship, 0.2–0.299, a weak relationship; and 0–0.199 implies that the variables are likely to be unrelated, even if significant *p*-values are encountered. In contrast, a low negative Kendall's tau b value approaching its minimum of −1.0 indicates that high rating of one endpoint is associated with low rating on another.

Statistical significance was set at *p* < 0.001 to highlight the most important results.

## Results

### Sample Details

Table 1 shows the details of the study sample. In total, 10,025 respondents completed the online survey. Of these, about 73% were females. A total of 70% of the respondents had completed tertiary level in terms of educational qualification. About 70% of the sample was from north Italy, 17% from central regions, 10% from the south, and only 72% were nationals living abroad. Further, 100% of the sample had good knowledge of the disease.

### Psychological Impact

**Figure 1:**
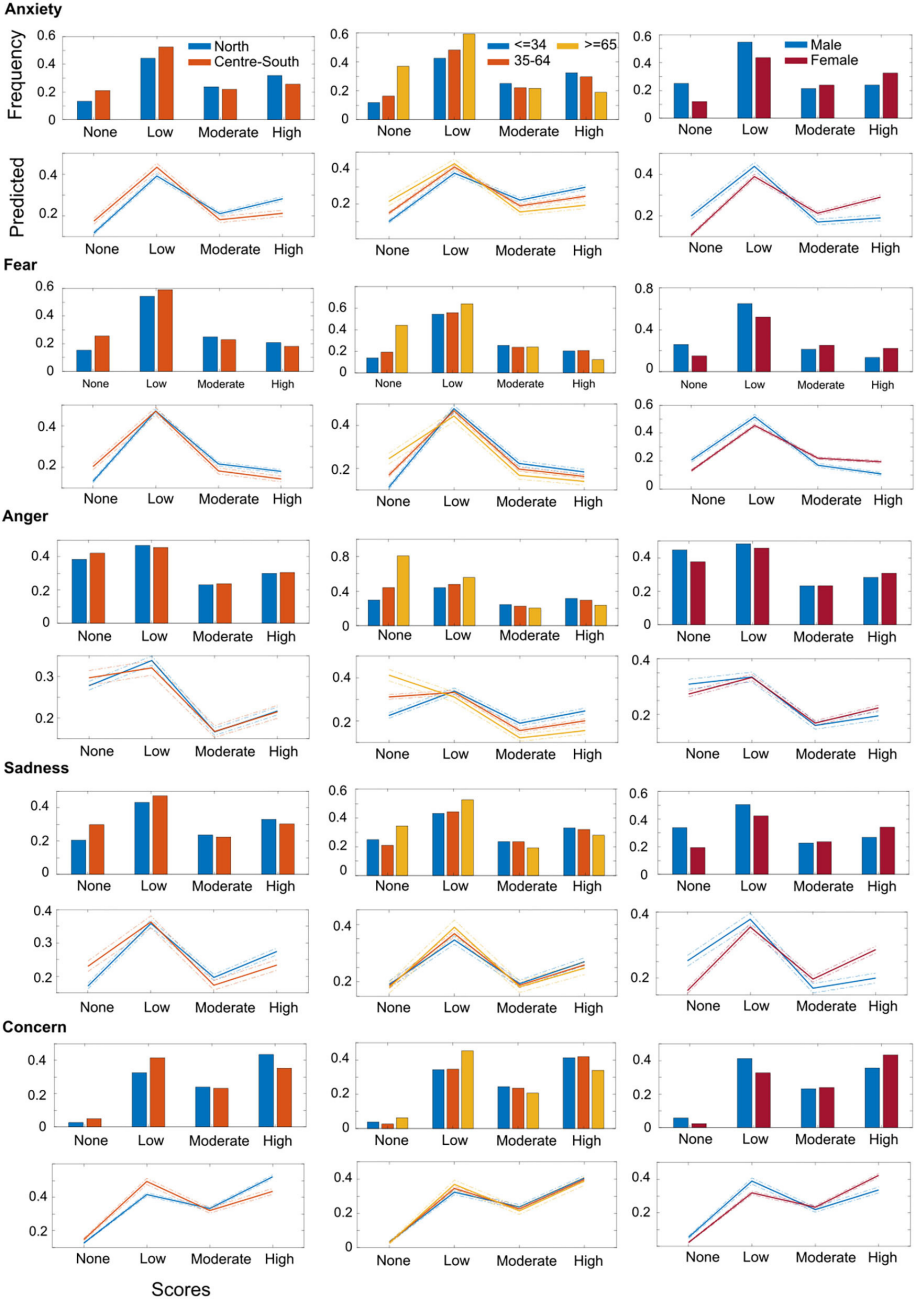
Multinomial logistic regression data for psychological impact (anxiety, fear, anger, sadness, concern) score (none, low, moderate, high) and demographic factors (Region, Age, and Gender). Top Panel: bars represent the frequency count of scores normalized over the total number of each demographic factor. Bottom panel: predicted category counts (marked line) for the multinomial logistic regression model and 95% confidence bounds.

Multinomial logistic regression determined the relationship between demographic factors of region, age, and gender and scores (none, low, moderate, high) obtained from the psychological impact category (anxiety, fear, anger, sadness, concern) (Figure 1 and Table 2).

**Table 2:** Multinomial logistic regression data for psychological impact category and demographic factors (region, age, and gender).

	**Beta**	**StdErr**	***t*-Stat**	***P*-value**
**REGION**				
Anxiety	0.32	0.03	9.42	**<0.0001**
Fear	0.27	0.03	7.84	**<0.0001**
Anger	0.02	0.03	0.73	0.4619
Sadness	0.21	0.03	6.19	**<0.0001**
Concern	0.33	0.03	9.36	**<0.0001**
**AGE**				
Anxiety	0.26	0.028	9.43	**<0.0001**
Fear	0.19	0.028	6.89	**<0.0001**
Anger	0.26	0.027	9.83	**<0.0001**
Sadness	0.03	0.027	1.26	0.2081
Concern	0.04	0.029	1.29	0.1979
**GENDER**				
Anxiety	−0.48	0.03	−13.96	**<0.0001**
Fear	−0.50	0.03	−14.22	**<0.0001**
Anger	−0.13	0.03	−3.97	**0.0001**
Sadness	−0.39	0.03	−11.61	**<0.0001**
Concern	−0.34	0.03	−9.68	**<0.0001**

*Significant P values are highlighted in bold*.

A significant relationship (*p* < 0.0001) was found between *Region* and anxiety, fear, sadness, and concern, but not with anger, and between *Region* and all *Daily Life* aspects that were evaluated. Data from north Italy exhibited higher prevalence of high psychological impact (anxiety 28%, fear 18%, anger 21%, sadness 27%, concern 42%) than the center-south zones (anxiety 21%, fear 14%, anger 22%, sadness 23%, concern 34%).

A significant relationship was found between *Age* and anxiety, fear, and anger, but not with sadness and concern (Figure 1). The highest prevalence of high psychological impact was in the <34 years' age group (anxiety 29%, fear 18%, anger 24%, sadness 27%, concern 40%), and 35–64 years' age group. The lowest prevalence was in the >65 years' age group (anxiety 14%, fear 9%, anger 14%, sadness 21%, concern 32%).

Regarding *Gender*, we found a significant relationship with all the emotions. The highest prevalence of high psychological distress was among females (anxiety 30%, fear 19%, anger 22%, sadness 29%, concern 42%).

Logistic regression determined the relationship between demographic factors (*Region, Age, Gender*) and *Daily Life* aspects and *Frightening Events*, which showed that the epidemic negatively influenced all daily life activities (Table 3). People, especially females, were afraid of economic crises, falling sick, and dying (Table 4).

**Table 3:** Multinomial logistic regression data for daily life activity category and demographic factors (region, age, and gender).

	**Beta**	**StdErr**	***t*-Stat**	***P*-value**
**REGION**				
Nutrition	0.42	0.04	10.32	**<0.0001**
Sense of freedom	0.48	0.04	13.19	**<0.0001**
Relationship with others	0.48	0.03	13.54	**<0.0001**
Sexuality	0.42	0.04	10.88	**<0.0001**
Sleep	0.37	0.04	9.38	**<0.0001**
**AGE**				
Nutrition	0.12	0.03	4.03	**0.0001**
Sense of freedom	0.23	0.03	7.92	**<0.0001**
Relationship with others	0.09	0.03	3.39	**0.0007**
Sexuality	0.24	0.03	8.04	**<0.0001**
Sleep	−0.03	0.03	−0.89	0.3722
**GENDER**				
Nutrition	−0.37	0.04	−9.39	**<0.0001**
Sense of freedom	−0.23	0.04	−6.27	**<0.0001**
Relationship with others	−0.16	0.03	−4.60	**<0.0001**
Sexuality	−0.01	0.04	−0.19	0.8469
Sleep	−0.41	0.04	−10.55	**<0.0001**

*Significant P values are highlighted in bold*.

**Table 4:** Multinomial logistic regression data for frightening events category and demographic factors (region, age, and gender).

	**Beta**	**StdErr**	***t*-Stat**	***P*-value**
**REGION**				
Falling sick	0.13	0.04	3.23	**0.0012**
Economic crises	0.14	0.04	3.17	**0.0015**
Job loss	0.1	0.03	3.67	**0.0002**
Death	0.11	0.03	2.92	**0.0034**
Psychosis	0.31	0.03	10.25	**<0.0001**
Quarantine	0.19	0.03	4.99	**<0.0001**
None	0.01	0.05	0.16	0.8707
**AGE**				
Falling sick	−0.11	0.03	−3.70	**0.0002**
Economic crises	0.09	0.03	2.95	**0.0031**
Job loss	0.26	0.03	8.59	**<0.0001**
Death	−0.07	0.02	−2.51	**0.0119**
Psychosis	0.09	0.03	2.35	**0.0186**
Quarantine	0.22	0.02	7.59	**<0.0001**
None	−0.14	0.04	−3.08	**0.0020**
**GENDER**				
Falling sick	−0.34	0.03	−8.90	**<0.0001**
Economic crises	−0.13	0.04	−3.15	**0.0016**
Job loss	−0.16	0.03	−4.36	**<0.0001**
Death	−0.38	0.03	−10.30	**<0.0001**
Psychosis	−0.04	0.03	−1.07	0.2839
Quarantine	−0.19	0.03	−5.19	**<0.0001**

*Significant P values are highlighted in bold*.

**Figure 2:**
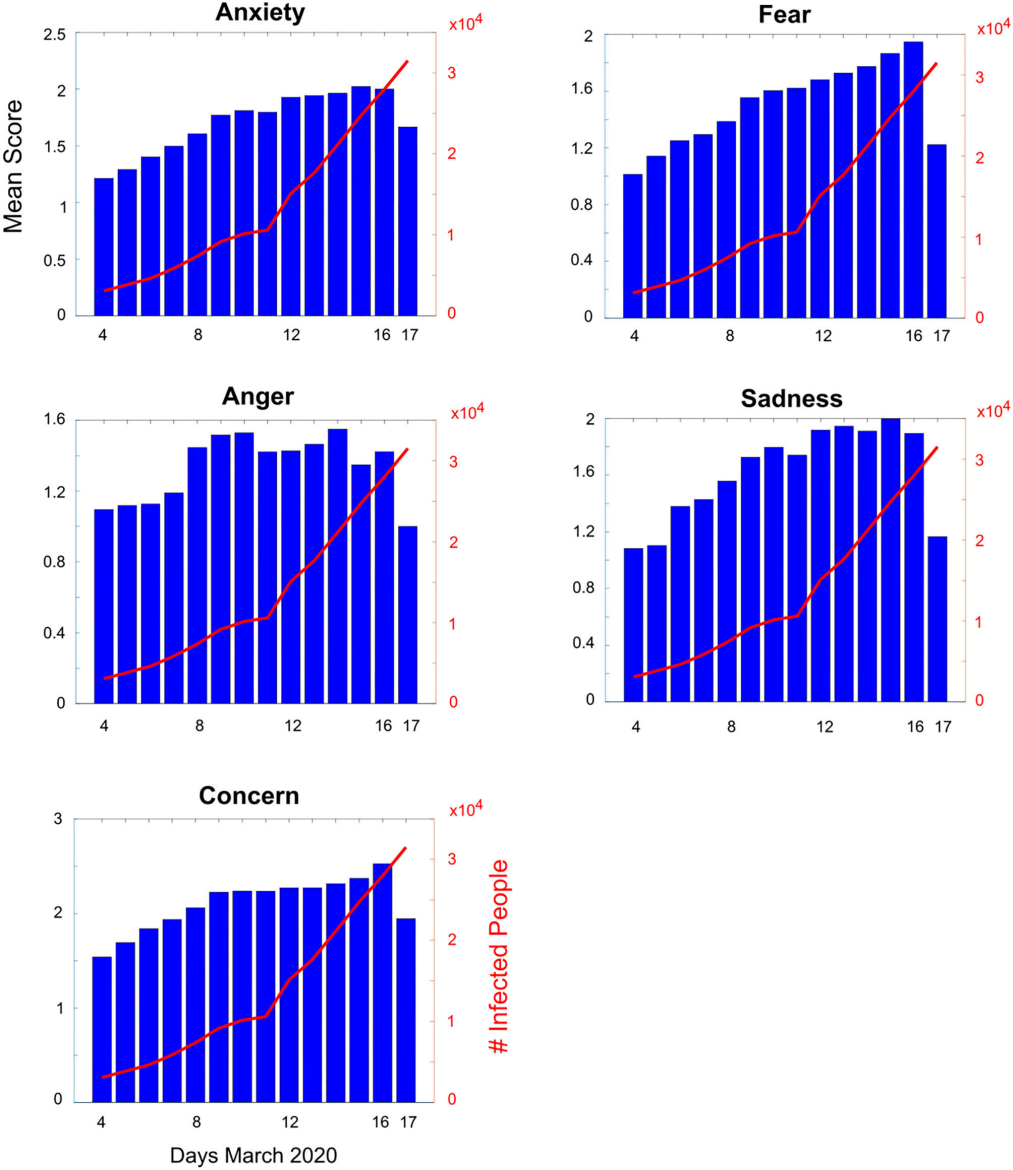
Correlation between the perceived psychological impact and increase in the number of COVID-19 cases (from 4 to 18 March 2020). Blue bars represent the mean score for psychological impact (anxiety, fear, anger, sadness, concern) per day. Red axis shows the increasing number of infected people during the same days.

We investigated the correlation between perceived psychological impact and increase in the number of COVID-19 cases. We found that “concern” and “sadness” had the strongest correlation values (tau = 0.23 and 0.22, respectively) (Figure 2). For *Daily Life*, all correlations were positive. “Sexuality and nutrition” showed the highest values (tau = 0.30 and 0.29, respectively). Regarding *Frightening Events*, the highest correlation value was found for “*Falling Sick* (tau = 0.15),” indicating a general uncorrelated trend in this category.

We finally investigated how different levels of psychological impact were correlated using pairwise comparison (Table 5).

**Table 5:** Correlation between increase in the number of COVID-19 cases and psychological impact, Daily Life Activity and Frightening Events categories.

Increase in the number of COVID-19 cases	**Psychological impact**										
	**Fear**		**Anxiety**		**Anger**		**Concerns**		**Sadness**		
	Kendall's Tau	*P*-value	Kendall's Tau	*P*-value	Kendall's Tau	*P*-value	Kendall's Tau	*P*-value	Kendall's Tau		*P*-value
	0.21	**<0.0001**	0.20	**<0.0001**	0.11	**<0.0001**	0.23	**<0.0001**	0.22		**<0.0001**
	**Daily life activity**										
	**Sleep**		**Nutrition**		**Sexuality**		**Relationship with others**		**Sense of freedom**		
	Kendall's Tau	*P*-value	Kendall's Tau	*P*-value	Kendall's Tau	*P*-value	Kendall's Tau	*P*-value	Kendall's Tau		*P*-value
	0.24	**<0.0001**	0.28	**<0.0001**	0.30	**<0.0001**	0.28	**<0.0001**	0.23		**<0.0001**
**Frightening events**											
**Psychosis**		**Economic crises**		**Falling sick**		**Death**		**Quarantine**		**Job loss**	
Kendall's Tau	*P*-value	Kendall's Tau	*P*-value	Kendall's Tau	*P*-value	Kendall's Tau	*P*-value	Kendall's Tau	*P*-value	Kendall's Tau	*P*-value
0.05	**<0.0001**	0.06	**<0.0001**	0.15	**<0.0001**	0.14	**<0.0001**	−0.003	0.072	0.03	0.00021

*Significant P values are highlighted in bold*.

Most correlations relating to the endpoints' relevance to *Psychological impact, Daily Life*, and *Frightening Events* were statistically significant. Tables 6 and 7 show low-to-moderate correlations (Kendall's tau statistic) except for *Falling Sick and Death*. These were issues most strongly associated with fear, anxiety, concerns, but not with anger or sadness.

**Table 6:** Correlations values (Kendall's tau statistics) relating to “psychological impact and daily life activity” categories.

**Daily life activity**	**Psychological impact**									
	**Fear**		**Anxiety**		**Anger**		**Concerns**		**Sadness**	
	**Kendall's Tau**	***P*-value**	**Kendall's Tau**	***P*-value**	**Kendall's Tau**	***P*-value**	**Kendall's Tau**	***P*-value**	**Kendall's Tau**	***P*-value**
Sleep	0.37	** <0.0001**	0.39	** <0.0001**	0.19	** <0.0001**	0.32	** <0.0001**	0.29	** <0.0001**
Nutrition	0.33	** <0.0001**	0.35	** <0.0001**	0.19	** <0.0001**	0.30	** <0.0001**	0.28	** <0.0001**
Sexuality	0.29	** <0.0001**	0.30	** <0.0001**	0.17	** <0.0001**	0.27	** <0.0001**	0.25	** <0.0001**
Relationship with others	0.37	** <0.0001**	0.39	** <0.0001**	0.19	** <0.0001**	0.38	** <0.0001**	0.29	** <0.0001**
Sense of freedom	0.32	** <0.0001**	0.36	** <0.0001**	0.21	** <0.0001**	0.35	** <0.0001**	0.30	** <0.0001**

*Significant P values are highlighted in bold*.

**Table 7:** Correlations values (Kendall's tau statistics) relating to “psychological impact and frightening events” categories.

**Frightening events**	**Psychological impact**									
	**Fear**		**Anxiety**		**Anger**		**Concerns**		**Sadness**	
	**Kendall's Tau**	***P*-value**	**Kendall's Tau**	***P*-value**	**Kendall's Tau**	***P*-value**	**Kendall's Tau**	***P*-value**	**Kendall's Tau**	***P*-value**
Psychosis	0.14	** <0.0001**	0.18	** <0.0001**	0.19	** <0.0001**	0.16	** <0.0001**	0.18	** <0.0001**
Economic crises	0.18	** <0.0001**	0.20	** <0.0001**	0.17	** <0.0001**	0.23	** <0.0001**	0.20	** <0.0001**
Falling sick	0.49	** <0.0001**	0.45	** <0.0001**	0.19	** <0.0001**	0.46	** <0.0001**	0.29	** <0.0001**
Death	0.49	** <0.0001**	0.42	** <0.0001**	0.19	** <0.0001**	0.39	** <0.0001**	0.28	** <0.0001**
Quarantine	0.24		0.25		0.16		0.20		0.20	
Job loss	0.17	** <0.0001**	0.18	** <0.0001**	0.16	** <0.0001**	0.18	** <0.0001**	0.17	** <0.0001**

*Significant P values are highlighted in bold*.

For *Psychological precautionary measure*s, the highest correlation value was found only for *Use of Medical Device* (Fear tau = 0.24; Anxiety tau = 0.21; Concern tau = 0.2), indicating a general uncorrelated trend in this category (Table 8).

**Table 8:** Correlations values (Kendall's tau statistics) relating to “psychological impact and psychological precautionary measure” categories.

**Psychological precautionary measure**	**Psychological impact**									
	**Fear**		**Anxiety**		**Anger**		**Concerns**		**Sadness**	
	**Kendall's Tau**	***P*-value**	**Kendal Tau**	***P*-value**	**Kendall's Tau**	***P*-value**	**Kendall's Tau**	***P*-value**	**Kendall's Tau**	***P*-value**
Use of medical device	0.24	** <0.0001**	0.21	** <0.0001**	0.11	** <0.0001**	0.21	** <0.0001**	0.15	** <0.0001**
Use of disinfectants	0.17	** <0.0001**	0.14	** <0.0001**	0.06	** <0.0001**	0.16	** <0.0001**	0.09	** <0.0001**
Listening to less drastic media information	−0.14	** <0.0001**	−0.10	** <0.0001**	0.01	0.09	−0.15	** <0.0001**	−0.07	** <0.0001**
Avoiding crowded places	0.13	** <0.0001**	0.13	** <0.0001**	0.006	0.51	0.16	** <0.0001**	0.09	** <0.0001**
Psychotherapy	0.17	** <0.0001**	0.19	** <0.0001**	0.09	** <0.0001**	0.13	** <0.0001**	0.15	** <0.0001**

*Significant P values are highlighted in bold*.

## Discussion

The emergency caused by the COVID-19 pandemic puts a strain on psychological health. This is the first study conducted in Italy that collected psychological data before government restrictions were imposed and during the quarantine period.

This study shows that people feel psychologically vulnerable and are afraid of economic crises, falling sick, and dying. Additionally, the psychological impact of the disease influences important daily life activities, such as sexuality, nutrition, sleep, and sense of freedom.

Factors associated with high psychological impact included female gender and young age. Scientific evidence suggests that psychological distress is less during mid-life and greater among younger people (12). This may be because young adults frequently engage with social media and may be more exposed to misinformation online, which can trigger psychological distress (13, 14). These results confirmed that younger people and woman are particularly vulnerable and have lower coping ability to deal with the consequences of this pandemic (15).

During the epidemic period, both before and after the lockdown, negative feelings experienced by people contributed to decreased psychological well-being (e.g., decreased sexuality, sleep disturbances, and nutrition-related issues).

There is a longstanding acceptance that psychological distress in the form of anxiety, sadness, irritability, self-consciousness, and emotional vulnerability is strongly correlated to physical morbidity (16). However, few studies investigated whether these negative feelings affect sexuality during a pandemic. In line with our results, Panzeri et al. (17) showed that the negative aspects of lockdown can affect the quality of sexual life, while Luetke et al. (18) showed that Americans experienced more conflict in their romantic partnerships, owing to changes in their intimate and sexual lives.

Sexual health is an important parameter for well-being because it impacts our psychological and emotional state. Sexual activities and orgasms serve as anti-depressants because these release certain hormones like oxytocin (the hormone that controls attachment), endorphin (the hormone related to well-being which helps to manage pain), and serotonin (the happiness hormone that works against anxiety) (19, 20).

Live statistics and COVID-19-related news tracking the number of confirmed cases, recovered patients, and death toll heighten concerns and uncertainty among populations. Health officials in a growing number of countries are fighting to slow down the spread of the novel virus and are also working toward curbing a secondary issue that the World Health Organization (WHO) calls “infodemic” (4). The WHO defines infodemic as “an overabundance of information—some accurate and some not—that makes it hard for people to find trustworthy sources and reliable guidance when they need it.” This problem is intensified by the ease and speed with which information can spread on social media. It generates fear and panic due to unverified rumors and exaggerated claims (6, 7, 15). It also promotes xenophobic and racist forms of digital vigilantism and scapegoating (16).

Only with responsible information can the concerns and uncertainty experienced by the whole community be addressed, ensuring that people avoid indulging in uncontrolled and risky behavior during an epidemic (e.g., xenophobia) (21).

Since the disease originated in China, Asia-phobic reactions have been reported at the beginning of the epidemic, in various regions of the world (18). Our results revealed that Italian people tend to engage in avoidant behavior toward people with pneumonia-like symptoms rather than toward Asians. This maybe because with the virus' spread throughout Europe, Italians may also be at the risk of being discriminated against.

Given that xenophobia during outbreaks is not uncommon, facing prejudice, including discrimination related to COVID-19, may add to feelings of isolation (18) and adversely affect mental health.

## Limitations

The strengths of this study lie in the large sample size, its extensive geographic coverage across Italy, and the early post-outbreak study period, but it has several limitations. Since we used an online survey, it is likely that the findings of the study under-represented the responses of those within certain demographics (e.g., those who are less educated, less affluent, and older respondents). Not everybody has access to the internet; online survey methodology is relatively uncontrolled, and the results are less generalisable.

We used a non-validated clinical questionnaire. The self-reported psychological impact may not adequately represent the mental health status assessed. Clinical prospective studies are necessary to provide more accurate data to support the need for focused public mental health strategies. This was a cross-sectional study, and associations between psychological impact and risk factors cannot be considered causal relationships.

The survey provides information on only the immediate psychological impact at a certain point of time. A longitudinal study is required to provide information on whether the observed impact would last for longer periods of time.

Additionally, those with a higher level of distress were probably more motivated to respond to the survey. Therefore, the extent of this response bias in the data cannot be accurately estimated.

The sample is far from being representative and consists mostly of individuals who accomplished higher levels of education, and it leans toward the female gender. Those who responded could be more inclined toward an interest in COVID-19-related information, and the sample could be biased due to the “infodemic.”

## Conclusion

This study determined the subjective psychological impact of Italy's first outbreak of COVID-19 on a substantial sample of the population. More than a quarter of the sample reported high levels of psychological impact that might require some form of external intervention. Certain groups, such as female and younger people are more vulnerable, and different aspects of well-being are impacted.

To support individuals in staying healthy during self-quarantine and isolation, we suggest a set of general tips (not based on the data). Using tele-health services is a valuable way of maintaining both physical and psychosocial health (22, 23). Staying virtually connected with friends and family and sharing emotions helps release any anxiety that one may have because it also helps improve communication among people (24). Staying updated with accurate health-related information and preventive measures could be associated with lower psychological impact (21). Feelings of stress and anxiety might only worsen if one closes oneself off from physically connecting with the significant other (19, 20).

Despite several limitations, our study provides information about the immediate psychological (emotional feelings) responses of Italy's general population to the COVID-19 pandemic. The survey covers several factors that can influence mental health. Our results help gauge the psychological burden on the community and suggest ways to minimize the impact.

## Data Availability Statement

The raw data supporting the conclusions of this article will be made available by the authors, without undue reservation.

## Ethics Statement

Ethical review and approval was not required for the study on human participants in accordance with the local legislation and institutional requirements. Written informed consent to participate in this study was provided by the participants' legal guardian/next of kin.

## Author Contributions

RF and AP were involved in all aspects of the research project, design, conducting the research, data handling, exploratory analysis, and drafting and editing the paper. MR, DM, MD, FR, FM, SB, BP, and GP assisted with the design of the study and in data interpretation and editing of the paper. AA conducted the statistical analysis and contributed to the drafting and review of the paper. All authors contributed to the article and approved the submitted version.

## Conflict of Interest

The authors declare that the research was conducted in the absence of any commercial or financial relationships that could be construed as a potential conflict of interest.
